# IPS in Supported Housing: Fidelity and Employment Outcomes Over a 4 Year Period

**DOI:** 10.3389/fpsyt.2020.622061

**Published:** 2021-01-14

**Authors:** Diana Roeg, Lars de Winter, Cris Bergmans, Chrisje Couwenbergh, Peter McPherson, Helen Killaspy, Jaap van Weeghel

**Affiliations:** ^1^Research Department, Kwintes Supported Housing, Zeist, Netherlands; ^2^Academic Center Mental Health, Tranzo, Tilburg University, Tilburg, Netherlands; ^3^Phrenos Center of Expertise for Severe Mental Illness, Utrecht, Netherlands; ^4^Division of Psychiatry, University College London, London, United Kingdom

**Keywords:** employment, IPS, supported housing, severe mental illness, fidelity

## Abstract

**Background:** People with severe mental illness have difficulties finding and maintaining competitive employment. This is particularly so for those living in supported housing who, by definition, have significant day-to-day support needs: in the Netherlands only 3 to 5% of people with serious mental health problems who live in supported housing are competitively employed. To support these people in finding and maintaining competitive employment, Individual Placement, and Support (IPS) was introduced within supported housing services in the Netherlands in 2015. As this is the first country that broadly implemented IPS in supported housing settings, this paper will focus on the first results regarding feasibility and effects on employment in clients of IPS in this sector.

**Methods:** We investigated the feasibility and employment outcomes of delivering IPS in supported housing services using fidelity assessments and quarterly employment outcomes on IPS program level within eight supported housing organizations, and compared these with 21 mental health treatment organizations in the Netherlands over a 4 year period. We investigated possible reasons for our findings and their implications through qualitative evaluations of the IPS fidelity assessors' notes and additional focus groups with IPS specialists and coordinators from supported housing services and fidelity assessors.

**Results:** The overall fidelity scores indicated reasonable implementation of the IPS model within both supported housing services and mental health services. However, there were differences between services with regard to specific fidelity items; mental health treatment organizations scored higher for team integration, whereas supported housing services scored higher for rapid job search and caseload size, diversity of jobs, and employers. Our qualitative data suggested that the difference in team integration between the two sectors was due to differences in their organizational and financial structures, as well as in the specific needs of their clients. Conversely, supported housing services had better connections with employers which facilitated more rapid job searching and greater diversity in employment opportunities. The average total client employment rate did not significantly differ; and was 25.8% per quarter in supported housing services and 29.6% in mental health treatment services.

**Conclusion:** Implementing IPS in supported housing settings is both feasible and effective.

## Introduction

People with severe mental illness face particular difficulties with meaningful social participation; research suggests that factors such as hospitalization, time spent in therapy, stigma and a lack of relevant skills, experiences, and educational opportunities, may limit the capacity and opportunity of individuals with severe mental illness to participate in valued social activities (1–3). This situation is worse for people with serious mental health problems living in supported housing services, who, by definition, have higher support needs with regard to activities of daily living and interpersonal skills and are at greater risk of social exclusion (1, 4–7). Employment is a key factor in the social recovery of persons with mental health problems; being in competitive employment can have numerous advantages for clients, beyond financial independence, such as improved mental health, self-esteem, personal recovery, and quality of life (8, 9). However, employment rates are lower amongst persons with severe mental illness compared to the general population. National surveys in the Netherlands have found that only 10–17% of clients with severe mental illness under the care of mental health services were competitively employed, with no indication that this situation is improving over time (10–12). For clients living in supported housing, the employment rate is even lower; despite there being no differences with clients from mental health treatment services in financial disincentives for working in terms of any impact on the individual's welfare benefits, only 3–5% of this group are reported to be competitively employed (5). These data highlight the need for broader implementation of vocational rehabilitation and supported employment services within the Netherlands, specifically targeting people with severe mental illness living in supported housing settings.

Individual Placement and Support (IPS) is a specific model of supported employment, developed to assist people with mental health problems find and maintain competitive employment (13). The model involves embedding a specific employment specialist within a community mental health team, and follows eight basic principles: the goal of competitive employment for clients; zero exclusion, and eligibility based on client choice; attention to client preferences; rapid job search; integration of employment services and mental health treatment; personalized welfare benefits counseling; targeted job development; and individualized, long-term support. IPS has been implemented in many parts of the world, across North America, Europe, Asia, and Australia, and its effectiveness has been extensively investigated (14). Numerous systematic reviews and meta-analyses have demonstrated the superiority of IPS over traditional vocational services, across multiple employment outcomes [see (15–19)]. In the Netherlands, a 30-month randomized controlled trial demonstrated the positive effects of IPS, with the intervention leading to greater improvements in employment outcomes for people with severe mental illness, when compared to other vocational services (9). Until recently, IPS had primarily been implemented within mental health treatment settings in the Netherlands; since 2015, however, it has also been implemented in supported housing services. Offering IPS in this setting extends the accessibility of evidence based vocational services, and complements existing vocational services already provided by supported housing staff.

In the current study, we examined IPS model fidelity and employment outcomes in supported housing services and mental health treatment services. Additional qualitative investigations were conducted to assist interpretation of the findings. The main research questions were: 1. How is IPS implemented in supported housing services, compared to mental health treatment services? 2. How are differences in the implementation of IPS between supported housing and mental health treatment services explained? 3. What are the employment outcomes in supported housing services?

## Methods

### Context

In the Netherlands, most clients (72.5%) of supported housing services receive floating outreach (ambulatory support), where support is provided in the service users own home (5). The remaining services offer accommodation-based support, typically organized as grouped apartments, with or without a shared living room and kitchen, and staff support available up to 24 h a day on-site (20, 21). In terms of the Simple Taxonomy for Supported Accommodation, these reflect Type 4 (individual accommodation, low/moderate support and no staff on-site), Type 2 and Type 3 services (congregate setting, high to moderate support, strong emphasis on move-on) (7). All service types have a strong emphasis on recovery and rehabilitation. Supported housing services provide support of varying intensity, addressing a range of service user needs including practical assistance with medication management, personal care, cooking, cleaning, and financial administration, and rehabilitative support to gain the skills and confidence to manage these tasks and to achieve personal goals in social and vocational domains (22, 23). Supported housing service users in the Netherlands are predominately male (65%), with a mean age between 44 and 50 years; approximately half have a primary diagnosis of a psychotic disorder (5, 22). Most supported housing clients (81%) receive additional treatment from mental health treatment services and, before moving to supported housing, most had either been hospitalized (36%) or living independently (43%) (22, 24). Any client with severe mental illness living in supported housing or receiving care from a mental health treatment organization who wishes to seek competitive work, is eligible for IPS.

### Sample

In the Netherlands, IPS outcome data and fidelity assessments have been collected nationally since 2016. IPS fidelity assessments are conducted by nine trained fidelity assessors, unaffiliated with the assessed IPS program, or organization. Program fidelity is assessed every 2 years and employment outcome data are collected every 3 months by the local IPS coordinator. The data are sent to the data coordinator of Phrenos Center of Expertise (LdW), who completes a quality check of accuracy, and consistency with the outcome reporting manual, before processing the data. For the current study, we analyzed data collected up to the end of 2019, including outcome and fidelity data from eight IPS programs within supported housing services, and 21 IPS programs within mental health treatment organizations.

### Measurements and Data Collection

#### Fidelity

During a full-day visit at the IPS program, two fidelity assessors conduct fidelity assessments according to the procedure described by Becker et al. (25). Data are collected from five different sources: interviews with IPS specialists, staff members, clients, family members and directors, observations of team meetings and vocational unit meetings, and review of program documents and client records. After the completion of the visit, assessors independently complete a 25-item fidelity rating scale (see below). Any rating discrepancies are discussed to achieve consensus ratings. Qualitative remarks are added for each item, when relevant, and programs receive a report with recommendations to help them improve quality.

IPS fidelity is assessed using the 25-item IPS fidelity scale (25). Each item is rated on a 5-point scale, ranging from 1 (no implementation) to 5 (full implementation), with intermediate numbers representing progressively greater degrees of implementation (25). The total score of the IPS fidelity scale is generated by summing all item scores, producing a total score ranging from 25 to 125 points. The scale developers defined benchmarks to assess descriptive labels regarding IPS fidelity (26). IPS programs scoring between 74 and 99 are considered to have ‘fair’ fidelity, programs scoring between 100 and 114 have “good” fidelity and programs scoring between 115 and 125 have “exemplary” fidelity. IPS programs scoring below 74 are considered to provide “no IPS,” indicating that IPS was not implemented in accordance with the model (25). The IPS-25 scale has good internal consistency (α = 0.88) and moderate predictive validity (*r* = 0.34) (26). Previous reports indicate that fidelity scores are positively associated with employment outcomes, and that improvement in fidelity scores predict improvement of program-level employment outcomes over time (26, 27). Therefore, fidelity assessment is a crucial element for quality improvement of IPS services.

#### Program Characteristics and Employment Outcomes

Data relating to program characteristics and employment outcomes are collected using a Dutch translation of the IPS Quarterly Employment Reporting Form (28). The form is administered quarterly and allows for the collection of the following data: the number of clients that received IPS, the number of clients that were competitively employed; the number of clients that left IPS services, and; the number and full-time equivalent of all employment specialists working in the IPS program. Employment rates are considered to be the main program outcomes, and are calculated by dividing the total number of clients competitively employed during the quarter by the total number of clients on the IPS workers' caseload over the same time frame. The total caseload is a dynamic cohort with clients leaving and joining the program at variable times. In order to achieve consistency in the reporting and interpretation of each variable on the Employment Reporting Form, definitions are provided in a manual for use by all IPS programs.

### Data Management

Each IPS program initiated IPS fidelity reviews, and submitted outcome data, at different time points between 2016 and 2019; programs were also at varying stages of implementation. Therefore, we used the first outcome report and fidelity assessment of each IPS program as the baseline measurement for our longitudinal analysis of each outcome. As each IPS program commenced at a different date, the time span of the data available per program differed; very few programs were in operation for the entire 4 year period. As such, we were only able to collate an overview of program characteristics and employment outcomes over the first 3 years from the start of implementation of IPS.

#### Focus Groups

As described above, fidelity assessors are able to provide comments on each item of the IPS fidelity scale. We used thematic analysis to analyze these comments, with the intention of providing a more complete understanding of the context and reasoning that led to the fidelity scores. We also organized a focus group with IPS assessor trainers and employment specialists involved in IPS coordination within the eight supported housing services. In this focus group, we discussed the validity of our findings from the thematic analysis, and explored participants' experiences and challenges with IPS implementation in supported housing services, with particular emphasis on fidelity items that differed between the sectors. The focus group was co-facilitated by DR and LW, with one co-facilitator taking notes. The focus group was audio recorded.

#### Ethical Approval

As per national legislation and standards, including the Medical Research Involving Human Subjects Act, and the Netherlands Code of Conduct for Research Integrity, ethical approval was not required for this study. The research relied on secondary analyses of national available data; focus group participants provided informed consent to participate.

### Data-Analysis

#### Quantitative Data Analysis

##### Program Characteristics and Employment Outcomes

We performed descriptive analyses of program characteristics (i.e., the number of clients receiving IPS, the number of clients newly enrolled in IPS, the number of clients that ended IPS services and the number and full-time equivalent of all employment specialists in the IPS programs), and employment outcomes in the first 3 years after the start of implementation of IPS, for supported housing and mental health services separately. Differences between supported housing and mental health services for each year were analyzed using a one-way analysis of variance (ANOVA). We controlled for the assumptions of homoscedasticity and normality of residuals.

##### Fidelity

We also conducted descriptive analyses on all individual fidelity item scores and the total score for supported housing and mental health services separately. As explained above, we used the first fidelity assessment for all IPS programs, for reasons of comparability. We analyzed differences between supported housing and mental health services using a one-way analysis of variance (ANOVA); the alpha level was set at 0.10 due to the small number of supported housing services (8) vs. mental health services (21). Stevens (29) suggests that when small group sizes are involved it is necessary to adjust the alpha level to compensate and set a cut-off of 0.10 or 0.15 rather than the traditional 0.05.

#### Qualitative Data-Analysis

##### Fidelity Assessors' Notes

To understand more about the implementation of IPS in supported housing settings, and why aspects of the IPS approach may have differed from its implementation in mental health treatment organizations, we used thematic analysis (30) to analyze the remarks fidelity assessors made to substantiate their scores. Two researchers (LG, DR, & LW) independently read all assessor comments from the fidelity score forms, with a focus on items where fidelity scores differed between the two sectors. They labeled all factors explaining the fidelity scores as either a facilitator or barrier, according to the content of the assessors' notes. They also compared the assessor notes for each IPS service with the official fidelity criteria. The inter-rater reliability (κ) of the results was calculated and discrepancies were discussed to achieve consensus.

##### Focus Group

Two trained IPS fidelity assessors, and seven employment specialists involved in IPS coordination in six supported housing organizations, participated in the focus groups. Focus group data were analyzed by reviewing the notes relating to each fidelity item, and by listening and re-listening to the focus group recording to ensure the participant comments were written down and interpreted correctly. Thematic analysis was used to analyze the data (30). The co-facilitator (LW) performed an interrater check. Any identified discrepancies were discussed and double checked using the audio recording, until consensus was reached.

## Results

### Quantitative Data-Analysis

#### Program Characteristics

IPS programs provided by supported housing services employed fewer IPS specialists, had fewer new enrollments and smaller caseloads, compared with mental health services (Table 1). For example, by the third year of the programs, IPS programs in mental health treatment organizations employed 10 IPS specialists (on average), who worked with an average 156 clients; in comparison, IPS programs in supported housing services employed 5.5 IPS specialists (on average), who worked with an average of 43 clients. This equates to an average caseload 15.6 vs. 7.8 per IPS specialist respectively.

Table 1Program characteristics over time^a^.

**Table:** 

		**Number of clients within the IPS program**				Number of newly enrolled clients^*^				**Number of clients that ended IPS services**				**Number of IPS specialists employed in each IPS program**			
**Year**	**Quarter**	**Supported housing**		**Mental health**		**Supported housing**		**Mental health**		**Supported housing**		**Mental health**		**Supported housing**		**Mental health**	
		**M (SD)**	***N***	**M (SD)**	***N***	**M (SD)**	***N***	**M (SD)**	***N***	**M (SD)**	***N***	**M (SD)**	***N***	**M (SD)**	***N***	**M (SD)**	***N***
Year 1	1	30.22 (17.91)	9	89.55 (69.04)	20					3.00 (2.67)	8	8.23 (8.67)	13	4.75 (1.67)	8	8.50 (4.93)	12
	2	33.78 (21.19)	9	97.25 (60.20)	20	8.75 (8.63)	8	17.85 (16.52)	13	3.50 (4.04)	8	12.29 (15.68)	14	4.38 (1.92)	8	9.21 (4.21)	14
	3	39.56 (23.03)	9	100.42 (57.49)	19	7.38 (7.37)	8	13.29 (16.28)	14	2.38 (2.45)	8	11.67 (16.34)	15	4.75 (1.39)	8	8.38 (4.40)	16
	4	54.50 (27.33)	8	100.37 (63.68)	19	10.43 (9.93)	7	27.40 (27.59)	15	2.57 (2.70)	7	13.38 (16.26)	16	5.00 (2.52)	7	8.25 (4.64)	16
Mean total year 1		39.09 (23.32)	9	96.81 (61.73)	20	8.78 (8.33)	8	19.74 (21.46)	15	4.71 (1.81)	8	8.57 (4.47)	16	2.87 (2.92)	8	11.52 (14.51)	16

**Table:** 

**Mean differences (ANOVA) between supported housing and mental health in year 1**													
***F***	***df***	***p***	***F***	***df***	***p***	***F***	***df***	***p***	***F***	***df***	***p***
28.64	1	0.001	5.51	1	0.02	10.72	1	0.001	21.52	1	0.01

**Table:** 

		**Number of clients within the IPS program**				Number of newly enrolled clients^*^				**Number of clients that ended IPS services**				**Number of IPS specialists employed in each IPS program**			
**Year**	**Quarter**	**Supported housing**		**Mental health**		**Supported housing**		**Mental health**		**Supported housing**		**Mental health**		**Supported housing**		**Mental health**	
		**M (SD)**	***N***	**M (SD)**	***N***	**M (SD)**	***N***	**M (SD)**	***N***	**M (SD)**	***N***	**M (SD)**	***N***	**M (SD)**	***N***	**M (SD)**	***N***
Year 2	1	56.71 (35.80)	7	101.17 (68.59)	18	3.50 (5.36)	6	26.19 (36.46)	16	2.80 (3.11)	5	14.76 (18.89)	17	4.43 (2.51)	7	7.88 (4.96)	17
	2	57.57 (49.04)	7	115.33 (81.11)	18	11.86 (15.57)	7	30.76 (35.64)	17	4.20 (4.27)	5	15.88 (17.33)	17	5.29 (2.93)	7	9.06 (5.66)	17
	3	44.00 (33.63)	6	127.44 (91.29)	18	5.83 (6.52)	6	29.12 (29.73)	17	4.50 (5.65)	6	16.35 (17.17)	17	4.83 (2.79)	6	9.88 (5.32)	16
	4	47.67 (21.83)	3	144.78 (91.28)	18	13.33 (11.93)	3	30.33 (31.12)	15	6.33 (5.69)	3	18.88 (22.62)	16	7.67 (5.51)	3	11.33 (5.72)	15
Mean total year 2		52.48 (36.64)	7	122.18 (83.38)	18	8.14 (10.77)	7	29.11 (32.64)	17	4.26 (4.47)	6	16.43 (18.69)	17	5.22 (3.10)	7	9.48 (5.43)	17

**Table:** 

**Mean differences (ANOVA) between supported housing and mental health in year 2**													
***F***	***df***	***p***	***F***	***df***	***p***	***F***	***df***	***p***	***F***	***df***	***p***
15.06	1	0.000	8.70	1	0.004	7.87	1	0.006	12.62	1	0.001

**Table:** 

		**Number of clients within the IPS program**				Number of newly enrolled clients^*^				**Number of clients that ended IPS services**				**Number of IPS specialists employed in each IPS program**			
**Year**	**Quarter**	**Supported housing**		**Mental health**		**Supported housing**		**Mental health**		**Supported housing**		**Mental health**		**Supported housing**		**Mental health**	
		**M (SD)**	***N***	**M (SD)**	***N***	**M (SD)**	***N***	**M (SD)**	***N***	**M (SD)**	***N***	**M (SD)**	***N***	**M (SD)**	***N***	**M (SD)**	***N***
Year 3	1	43.67 (20.60)	3	143.88 (83.88)	16	0.33 (.58)	3	25.86 (33.91)	14	5.67 (6.03)	3	15.87 (17.94)	15	6.67 (5.51)	3	9.87 (6.30)	15
	2	43.00 (20.07)	3	150.33 (87.09)	15	5.67 (1.15)	3	21.86 (24.02)	14	6.00 (1.73)	3	19.13 (15.67)	15	7.00 (6.08)	3	10.31 (5.68)	13
	3	42.50 (38.89)	2	152.33 (91.51)	15	7.50 (10.61)	2	23.73 (30.87)	15	4.50 (3.54)	2	18.67 (17.09)	15	3.50 (.71)	2	9.93 (5.90)	15
	4	44.00 (42.43)	2	179.00 (110.36)	14	3.50 (4.95)	2	40.21 (38.65)	14	2.00 (1.41)	2	21.00 (14.60)	14	3.50 (.71)	2	10.64 (5.75)	14
Mean total year 3		43.30 (23.50)	3	155.80 (91.49)	16	4.00 (4.88)	3	47.84 (32.23)	15	4.80 (3.58)	3	18.63 (16.08)	15	5.50 (4.25)	3	10.18 (5.77)	15

**Table:** 

**Mean differences (ANOVA) between supported housing and mental health in year 3**													
***F***	***df***	***p***	***F***	***df***	***p***	***F***	***df***	***p***	***F***	***df***	***p***
14.67	1	0.000	5.39	1	0.023	7.24	1	0.009	5.96	1	0.017

a *M and SD are the means and standard deviations of the descriptives of IPS programs. N is the number of IPS programs of which data was available*.

* *Newly enrolled people could not be calculated for the first quarter after implementation because calculations are based on previous quarters and therefore not applicable for the first quarter after start of implementation*.

#### Employment Outcomes

**Figure 1:**
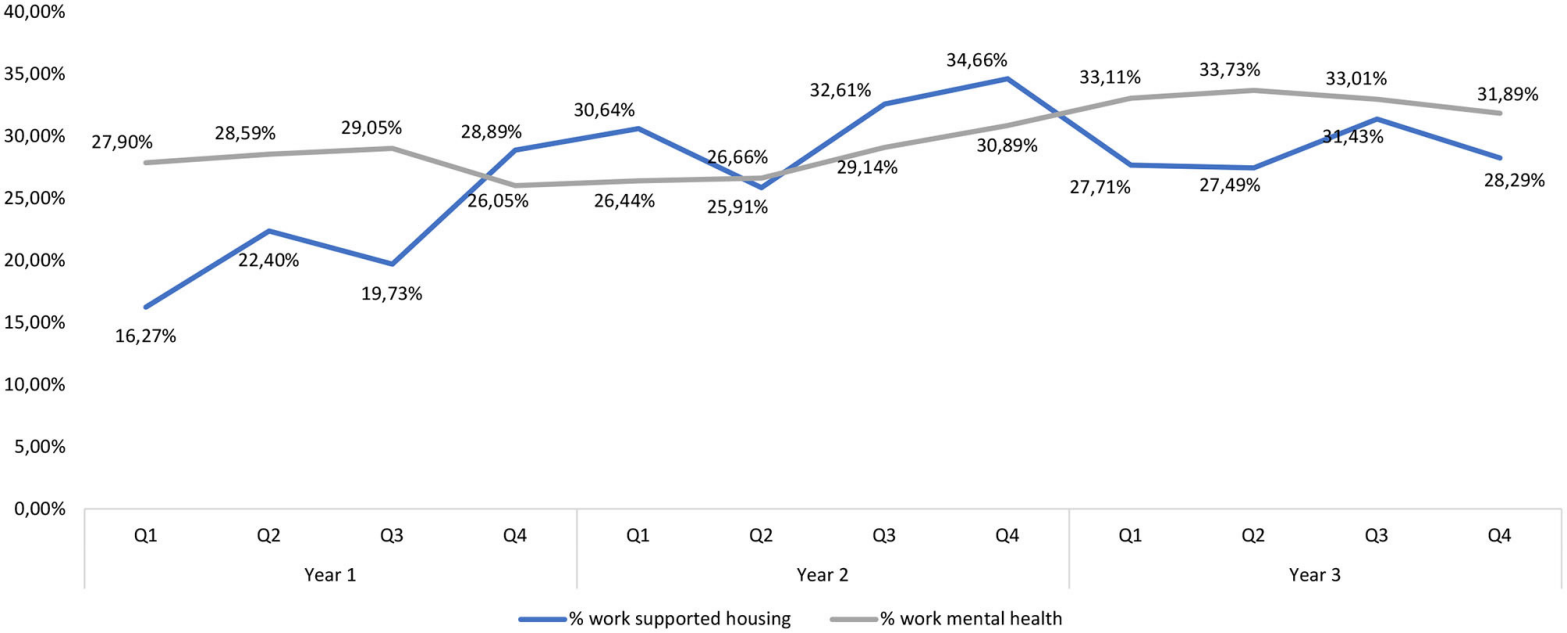
Employment rate as percentage of the total caseload of IPS programs in mental health and supported housing.

The quarterly employment rate for IPS programs in supported housing services was, on average, 25.8% of the total caseload and in the mental health treatment organizations this was 29.6%. There were no significant differences in employment rates between supported housing and mental health services in the first (*F* = 3.41; *df* = 1; *p* = 0.07), second (*F* = 0.63; *df* = 1; *p* = 0.43), and third year (*F* = 1.20; *df* = 1; *p* = 0.28) after the start of IPS (Figure 1).

#### Fidelity Assessment

The average IPS fidelity score in supported housing was 94.63, indicating “fair” implementation; this was not significantly different from the average score of mental health service provided IPS programs (M = 94.63; SD = 9.36 vs. M = 90.43; SD = 11.25; *F* = 1.23; *p* = 0.28; Table 2).

**Table 2:** Fidelity assessments mental health vs. supported housing organizations.

	**Supported housing**			**Mental health**			**Differences (ANOVA)**		
	**M**	**SD**	***N***	**M**	**SD**	***N***	***F***	***df***	***p***
Total score	94.63	9.36	8	90.43	11.25	21	1.23	1	0.28
**1. Staffing**									
Item 1. Caseload size^*^	4.75	0.71	8	3.76	1.45	21	3.37	1	0.08
Item 2. Employment services staff	4.75	0.71	8	3.76	1.64	21	2.15	1	0.15
Item 3. Vocational generalists	4.38	0.92	8	4.43	0.87	21	0.02	1	0.88
**2. Organization**									
Item 1. Integration through team assignment^**^	2.38	0.74	8	4.29	1.19	21	17.14	1	0.00
Item 2. Integration through frequent team contact^**^	2.25	1.04	8	3.88	1.05	21	11.06	1	0.00
Item 3. Collaboration between employment specialists and Vocational Rehabilitation counselors	3.75	0.46	8	3.95	0.80	21	0.02	1	0.88
Item 4. Vocational unit	3.50	1.20	8	3.00	1.34	21	0.41	1	0.53
Item 5. Role of employment supervisor	1.88	1.36	8	2.05	1.43	20	0.29	1	0.59
Item 6. Zero exclusion criteria	4.00	0.00	8	3.90	1.00	21	0.41	1	0.53
Item 7. Agency focus on competitive employment	3.00	1.20	8	3.02	0.93	21	0.03	1	0.87
Item 8. Executive team support for SE	3.50	1.41	8	2.84	1.50	19	0.61	1	0.44
**3. Services**									
Item 1. Work incentives planning	4.00	0.93	8	4.14	0.91	21	0.14	1	0.71
Item 2. Disclosure	4.38	0.92	8	4.76	0.62	21	0.22	1	0.64
Item 3. Ongoing, work-based vocational assessment	3.88	0.64	8	4.19	0.60	21	1.54	1	0.23
Item 4. Rapid search for competitive job^**^	4.00	0.76	8	2.52	1.29	21	7.58	1	0.01
Item 5. Individualized job search	4.38	0.92	8	4.71	0.46	21	1.26	1	0.27
Item 6. Job development—Frequent employer contact	2.50	0.76	8	2.38	1.16	21	0.02	1	0.88
Item 7. Job development—Quality of employer contact	3.13	1.13	8	3.38	1.28	21	0.33	1	0.57
Item 8. Diversity of job typesn^*^	5.00	0.00	8	3.81	1.72	21	4.15	1	0.05
Item 9. Diversity of employers^*^	5.00	0.00	8	3.76	1.81	21	4.02	1	0.06
Item 10. Competitive jobs	2.13	1.13	8	2.29	1.52	21	0.11	1	0.74
Item 11. Individualized follow-along supports	4.75	0.46	8	4.33	1.35	21	0.71	1	0.41
Item 12. Time-unlimited follow-along supports	4.63	0.52	8	4.00	1.45	21	1.39	1	0.25
Item 13. Community-based services	3.38	1.41	8	3.05	1.20	21	0.50	1	0.49
Item 14. Assertive engagement and outreach by integrated treatment team^**^	3.75	1.28	8	4.57	0.60	21	5.15	1	0.03

* p < 0.10/

** *p < 0.05*.

However, there were differences in some individual fidelity item scores. Mental health treatment organizations scored significantly higher on Items 1 and 2 of the organization section: “*Integration through team assignment”* and “*Integration through frequent team contact,”* and Item 14 of the services section: “*Assertive engagement and outreach by integrated treatment team*.” On the other hand, IPS fidelity was higher in supported housing services for Item 1 of the staffing section: “*Caseload*,” and items 4, 8, and 9 of the services section: “*Rapid search for competitive job,”* “*Diversity of job types,”* and “*Diversity of employers.”* See attachment for the total IPS-25 scale, including explanation for each item.

### Qualitative Data-Analysis

Below, we present possible explanations for the differences in individual fidelity score item, derived from thematic analyses of the IPS fidelity assessors' notes (see Table 3) and focus group data. In addition, results from the focus groups are presented here to explain, expand upon, and/or give context to the audit data. We calculated inter-rater reliability by coding the themes that were rated equally by both assessors with “1” and themes that needed consensus with “0”; this coding system allowed us to calculate inter-rater reliability. The inter-rater reliability of the analysis of the assessors notes between the assessors was moderate (κ = 0.56).

Table 3Thematic analysis of the seven fidelity items on which supported housing accommodations and mental health treatment institutes differed.

**Table:** 

**Analysis of themes in fidelity item**						
**Themes**	Number of times mentioned^*^		**Thematic scoring explained**	Facilitators (F) and barriers (B) counted^*^		**Selection of illustrative citations**
	**MH (*****N*** **=** **21)**	**SH (*****N*** **=** **8)**		**MH (*****N*** **=** **21)**	**SH (*****N*** **=** **8)**	
**Item 1. Caseload size**						
1. Number of clients per full-time equivalent	19	8	Following the anchor points for scoring of the fidelity review. Caseload per full-time equivalent ≤20: facilitator. Caseload per full-time equivalent ≥40: barrier	F: 9 B: 2	F: 8 B: 0	1. There are 20 or less clients per fulltime IPS specialist2. Based on the caseload reports provided, they support 75 IPS trajectories with two IPS specialists (1.56 full time equivalent). This comes down to a larger caseload than 41 clients/IPS specialist
2. Mixed caseload	3	1	Considered a barrier is mentioned as main reason for a low final score	F: 0 B: 3	F: 0 B: 0	Based on the total caseload of all IPS specialists in organization x, we concluded most IPS specialists work with (large) mixed caseloads
3. Proper registration time spent on IPS trajectories	2	0	Interpreted as facilitator or barrier accordingly to the assessor's interpretation	F: 0 B: 2	F: 0 B: 0	The time spent on IPS vs. other trajectories is not clearly defined and the caseload is difficult to interpret for the assessors
4. Working with waiting lists	1	0	Interpreted as facilitator or barrier accordingly to the assessor's interpretation	F: 0 B: 0	F: 0 B: 0	Advised is to make a plan for future waiting lists, as there is one already with one IPS specialist, and for others there is a risk on one
**Item 2. Integration of supported employment with mental health treatment through team assignment^a^**						
1. IPS specialist and caseload related to specific teams	21	8	Facilitator: At least 90% of the caseload of the IPS specialist belongs to one or two teams. Barrier: IPS specialist is not connected to specific teams	F: 15 B: 1	F: 0 B: 3	1. IPS specialists are connected to one or two mental health teams. 90–100% of the caseload is from these teams.2. None of the IPS specialists are structurally part of a mental health team
2. Combining functions	2	1	Not applicable^b^	F: 0 B: 0	F: 0 B: 0	1. The IPS specialists have often a combined function and a large caseload2. Furthermore, the IPS specialists provide all kinds of support to work as well as other activities for the clients of organization x. They have to divide their time
3. Large caseload	1	1	Not applicable	F: 0 B: 0	F: 0 B: 0	All IPS specialists are part of one location only and sometimes more than one. … Location size and teams differ largely from small (30 clients) to large (250 clients)
4. Working closely with mental health team members	2	2	Interpreted as facilitator or barrier accordingly to the assessor's interpretation	F: 0 B: 1	F: 1 B: 0	The IPS specialist discusses clients with the key social worker or behavioral expert on case level and joins team meetings where caseloads are discussed
5. Divers caseload and from different teams	5	3	Interpreted as facilitator or barrier accordingly to the assessor's interpretation	F: 0 B: 3	F: 0 B: 1	The other four IPS specialists are mainly working for two mental health treatment teams, but also have some additional IPS trajectories in other teams
6. Time restraints in providing IPS	2	1	Not applicable	F: 0 B: 0	F: 0 B: 0	A number of IPS specialists provide IPS trajectories for several teams and have limited time per team
**Item 3. Integration of supported employment with mental health treatment through frequent team member contact**						
2. Integration at intake	2	2	Interpreted as facilitators or barrier accordingly to the assessor's interpretation	F: 1 B: 0	F: 0 B: 0	IPS specialist is not present at intakes, leading to little opportunities to influence the team and enlarge attention and enthusiasm for IPS trajectories for new clients
3. Discussing caseload on a regular base	1	0	Interpreted as facilitators or barrier accordingly to the assessor's interpretation	F: 0 B: 0	F: 0 B: 0	The total caseload is discussed on a regular base

**Table:** 

**Analysis of presence of criteria**						
	**Mental Health**			**Supported housing**		
**Criteria**	***N*** **present**	**% present**	***N*** **total**	***N*** **present**	**% present**	***N*** **total**
1. Attends weekly client focused meetings	14	66.7%	21	0	0.0%	8
2. Participates actively in the team meetings	18	85.7%	21	2	25.0%	8
3. Employment services documentation (vocational assessment/profile, employment plan, progress notes) is integrated into the client's mental health record	13	61.9%	21	7	87.5%	8
4. Employment specialist's office is in close proximity to (or shared with) the mental health team members	19	90.5%	21	6	75.0%	8
5. Employment specialist helps the team think about employment for people who haven't yet been referred to supported employment services	19	90.5%	21	3	37.5%	8

**Table:** 

**Item 4. Rapid job search**						
1. Rapid employer's contact	10	8	First contact within a month is defined as facilitator.	F: 1 B: 0	F: 2 B: 0	The first employer's contact takes place within 31 and 60 days after start on average
2. Incomplete registration	15	2	Not applicable	F: 0 B: 0	F: 0 B: 0	The caseload report provided is not complete. The IPS trajectories of all IPS specialists are not clear for the assessors
3. Based on client's needs	1	0	Interpreted as facilitator or barrier accordingly to the assessor's interpretation	F: 0 B: 0	F: 0 B: 0	Employers contacts are based on clients' preferences concerning type of job (i.e., what do they like, personal goals) and needs (including experience, talent, symptoms, health, etcetera) instead of opportunities available (i.e., jobs available immediately)
4. Influence of IPS financing	1	0	Interpreted as facilitator or barrier accordingly to the assessor's interpretation	F: 0 B: 0	F: 0 B: 0	Furthermore, financing of IPS trajectories can determine the pace
5. Cases put on hold	1	0	Interpreted as facilitator or barrier accordingly to the assessor's interpretation	F: 0 B: 0	F: 0 B: 0	Quit a lot clients in the caseload were put “on hold” (26% on average). If this stays this way, due to treatment priorities, IPS specialists can replace these cases with active cases to make room for new entries
**Item 5. Diversity of job types**						
1. Diversity of job types	14	7	Counted as facilitator is in more than 85% of the clients a divers offer is noted by the assessor.	F: 9 B: 0	F: 7 B: 0	In 85–100% of the cases IPS specialists support clients in finding a diversity of jobs
2. <10 competitive jobs	5	0	Interpreted as facilitator or barrier accordingly to the assessor's interpretation	F: 0 B: 0	F: 0 B: 0	IPS specialists support clients in a diversity of jobs. If there are <10 paid jobs, the fidelity score is set on 1
3. Diversity related to the client's preferences	1	1	Interpreted as facilitator or barrier accordingly to the assessor's interpretation	F: 0 B: 0	F: 0 B: 0	The client's preferences are the starting point. … the IPS specialists work hard to find employment that fits these preferences. All kinds of available grants, contracts and schemes are used
4. Job search by third parties	1	0	Interpreted as facilitator or barrier accordingly to the assessor's interpretation	F: 0 B: 0	F: 0 B: 0	The job coaching is not always provided by the IPS specialists
**Item 6. Diversity of employers**						
1. Employers diversity	13	8	Interpreted as facilitator if in more than 85% of the clients employers diversity is noted by the assessor.	F: 8 B: 0	F: 7 B: 0	IPS specialists help clients in getting jobs by divers employers in 85–100% of the time. Jobs and trial placements are with different employers
2. <10 competitive jobs	5	0	Interpreted as facilitator or barrier accordingly to the assessor's interpretation	F: 0 B: 0	F: 0 B: 0	There are too little paid jobs in the caseload report to score this item. If there are <10 paid josbs, this item is scored with a 1
3. No regular or competitive employers	1	0	Interpreted as facilitator or barrier accordingly to the assessor's interpretation	F: 0 B: 0	F: 0 B: 0	A number of clients work in supported employment settings and earn the minimum wage. These contracts does not count as competitive employment as they do not consider regular jobs
4. Employers overview not available	1	0	Interpreted as facilitator or barrier accordingly to the assessor's interpretation	F: 0 B: 0	F: 0 B: 0	There is no report of different employers available. This item cannot be scored
5. Mediation by external parties	1	0	Interpreted as facilitator or barrier accordingly to the assessor's interpretation	F: 0 B: 0	F: 0 B: 0	Most clients are mediated by reintegration agencies
**Item 7. Assertive engagement and outreach**						
1. Once it is clear that the client no longer wants to work	4	0	Interpreted as facilitator or barrier accordingly to the assessor's interpretation	F: 0 B: 0	F: 0 B: 0	If it becomes clear a client does not want to work anymore, or does not want to make use of the IPS specialist, the outreach is stopped

**Table:** 

**Analysis of presence of criteria**						
	**Mental health**			**Supported housing**		
**Criteria**	***N*** **present**	**% present**	***N*** **total**	***N*** **present**	**% present**	***N*** **total**
1. Service termination is not based on missed appointments or fixed time limits.	21	100.0%	21	8	100.0%	8
2. Systematic documentation of outreach attempts.	15	71.4%	21	6	75.0%	8
3. Engagement and outreach attempts made by integrated team members.	20	95.2%	21	7	87.5%	8
4. Multiple home/community visits.	15	71.4%	21	6	75.0%	8
5. Coordinated visits by employment specialist with integrated team members	20	95.2%	21	7	87.5%	8
6. Connect with family, when applicable.	19	90.5%	21	6	75.0%	8

* *MH, Mental health treatment services; SH, Supported housing services*.

a *In supported housing accommodations integration was determined based on the integration through team assignment to a supported housing team (in comparison to mental health care teams)*.

b *Not applicable as it did not lead consequently to a negative either a positive influence on the fidelity score. These consider themes that are noted by the assessors and concern context information. In this way these themes can, but not always do influence the fidelity score*.

#### Higher Fidelity IPS Items in Mental Health Services

##### Integration Through Team Assignment

We compared the average score and assessors' notes for the item “*Integration through team assignment*” for the IPS programs in mental health and the IPS programs in supported housing. The score on this item is based on the number of teams that one IPS specialist is assigned to (on average) and the percentage of clients in the caseload that come from these assigned teams (i.e., not from multiple other referrals). The rationale is that with a low number of assigned teams, and by receiving most referrals via these teams, the integration of IPS services and treatment is higher. The thematic analysis showed that IPS specialists in mental health treatment more often serve one or two mental health treatment teams; conversely, IPS specialists in supported housing services were less integrated, often serving more than two teams. In the majority of the IPS programs within mental health treatment organizations, at least 90% of the caseload of individual IPS specialists was from one or two mental health treatment teams, compared with a minority of the IPS specialists working with supported housing services. In the focus group, IPS coordinators in supported housing services explained this finding as being due to the fact that clients living in supported housing tended to have more intensive support needs, and supported housing teams also tended to be smaller, with fewer clients per team than mental health treatment teams; as such, IPS specialists in these settings had to work across a number of locations or teams in order to achieve a full caseload (maximum of 20 clients).

An IPS specialist explained this as follows: “*That has also to do with the geographical spread and the number of clients. We are organized by self-organization and work in small teams of 8 to 12 team members. In our branch, [as an IPS specialist] you need at least five teams for your caseload.”*

##### Integration Through Frequent Team Contact

The thematic analysis of the IPS fidelity assessors' notes showed that IPS specialists were only able to fully integrate within team meetings in a relatively small number of supported housing services, whereas those working in mental health treatment organizations tended to attend the weekly mental health treatment team meetings more often, and actively participate in treatment team meetings more frequently. They also helped the team think about employment for people who hadn't yet been referred to supported employment services more often than IPS specialists working with supported housing services. In the focus groups, IPS specialists explained that due to the practical barriers for team assignment mentioned above, the contact intensity between IPS specialists with teams and their members, as well as active participation in meetings was lower in supported housing services. Most IPS specialists described this as a structural factor that was characteristic of the sector and could not be solved. Despite this, the IPS assessor acknowledged that in some supported housing organizations quite good integration was achieved. Difficulties with team integration exist for IPS specialists in supported housing services, when teams are small and located in rural areas so IPS specialists cannot fill their caseload by integrating with a maximum of two teams. Furthermore, participating in the regular team meetings was more difficult in some services than others. For instance, some supported housing services did not have a formal, regular team meeting. Some IPS specialists solved this by building on their personal contact with team members, and “being available” for the team when they had employment or education related queries about their clients; these steps served to increase their integration within the service.

The IPS fidelity assessor explained how this was addressed when measuring fidelity on integration, next to joining and integrating in team meetings and being located close by team locations*: “What is relevant is whether paid work is mentioned as a goal in the guidance plan. We ask about joining team meetings and how integration is sought in other ways with their colleagues involved by the client. … In that context, the contact with a person's main social worker is highly relevant, as this is the person that integrates the IPS goals in the broader guidance plan.”*

##### Assertive Engagement and Outreach by Integrated Treatment Team

The thematic analysis of the assessor notes did not identify any clear evidence to explain these findings. In the focus group, participants did not recognize the somewhat lower score in supported housing services, which would reflect fewer outreach attempts and more missed appointments, resulting in higher rates of discharge from IPS. They stated they would have expected supported housing services to score higher on this item than IPS specialists working in mental health services, as the first is primarily concerned with community living, rehabilitation and practical housing support. One participant suggested that the weekly mental health treatment team meetings might add to the early detection of employment needs in mental health treatment services, though the other participants did not agree.

#### Higher Fidelity IPS Items in Supported Housing Services

##### Caseload Size

Analysis showed that all IPS programs in supported housing services supported 20 or fewer clients per IPS specialist, which is the standard in for the IPS model. In contrast, less than half of mental health treatment organizations supported 20 or fewer clients per IPS specialist, with some IPS programs supporting more than 40 clients. In addition, IPS specialists within mental health treatment organizations more frequently worked with a “mixed caseload,” supporting clients in both IPS and other forms of (vocational) rehabilitation. In the focus group, these findings were explained: participants suggested that that, due to the different support needs of clients living in supported housing, IPS specialists required more time to work with each client, compared to IPS specialists working with clients in mental health services. Furthermore, participants indicated that supported housing services had more problems arranging funding for clients to access IPS, leading to reduced accessibility for supported housing clients. As a consequence, clients for which IPS funding was available were sometimes spread over a large geographical area, with IPS specialists requiring more traveling time.

An IPS coordinator mentioned: “*At our organization, we invested strongly in IPS. We wanted to be able to provide IPS to all clients that were interested. … However, we experienced on the way that not all IPS hours can easily be financed in our sector.”*

The IPS assessor explained that the most important funding scheme for both sectors includes the subsidy scheme from the Employee Insurance Agency commissioned by the Ministry of Social Affairs and Employment, and for persons receiving welfare payments, municipalities are financially responsible for vocational trajectories. Funding for the treatment and housing services is different: health insurers pay for treatment, while municipalities pay for supported housing services. As a consequence, in mental health treatment organizations, the first eight IPS contacts are often paid by insurers.

##### Rapid Search for Competitive Job

The thematic analysis of the fidelity assessors' notes showed that, compared with mental health treatment organizations, a higher percentage of IPS programs in supported housing services were able to arrange the initial face-to-face contact between client and employer within 1 month of program entry (4.8 vs. 25%, respectively). However, this finding may have been influenced by the fact that the assessors were less likely to be able to identify the time between first employer contact and program entry due to incomplete registrations in IPS services in mental health services than those delivered in supported housing services. In the focus groups, participants suggested that IPS specialists in supported housing services were able to invest more time into job searches due to lower caseloads, and often had contact with clients prior to starting with IPS and so knew at an early stage what kind of work clients were interested in.

An IPS specialist explained this further: “*In team x, I know all the clients, even though I do not coach them in their job search. As soon as they formulate a wish for work, and I am consulted, then I already know a little what his/her preferences are. I think this is helpful.”*

##### Diversity of Job Types and Diversity of Employers

The thematic analysis of the fidelity assessors' notes showed that the lower fidelity on these two items was due to IPS specialists in supported housing services being more able to support their clients into different types of jobs, as well as in different types of employers/companies. In all supported housing services, IPS specialists assisted clients in obtaining different types of jobs, while this was the case in only half of the mental health treatment organizations. Additionally, most of the supported housing services (85%) also worked together with several companies. Five IPS programs within mental health treatment services supported <10 clients into competitive employment, which automatically led to a score of one; this was not the case in any IPS programs in supported housing during the first fidelity assessment. Focus group participants suggested that supported housing services are, from origin, needs based and may have developed particular strengths in creatively searching for services and facilities that fit clients' needs. They indicated that this should also be the case in mental health treatment organizations.

An IPS coordinator simply stated: “*that is what you need to do in IPS.”*

## Discussion

### Summary of the Findings

In the current study, we analyzed fidelity scores of IPS programs in supported housing services in The Netherlands and compared them with fidelity scores of IPS programs in mental health treatment organizations. The purpose was to understand feasibility of IPS implementation in supported housing services, in order to increase the accessibility of evidence based vocational services for persons with severe mental illness. Employment rates were examined to understand the relative impact of the IPS programs. Results showed that overall IPS fidelity in supported housing services was “fair,” with services scoring, on average, 94.63; no significant difference was found in overall fidelity scores between supported housing, and mental health treatment organization, based IPS programs suggesting that IPS is feasible in both sectors. Item-level analyses of the IPS fidelity scale indicated that mental health treatment organizations demonstrated better integration of the IPS programs, and higher scores on engagement and outreach; conversely, IPS programs in supported housing services provided more rapid job search processes and more diverse selection of competitive work types and employers. IPS programs in supported housing services were also found to have smaller caseloads per IPS specialist than those provided in mental health treatment services. The quarterly employment rate for IPS programs in supported housing services was 25.8% on average, similar to the rate in mental health treatment organizations, suggesting that these are relatively successful programs; recent research suggests employment levels amongst supported housing clients in The Netherlands is typically 3–5% (5). The fidelity scores and employment rates reported in the current study are similar to longstanding averages reported for Dutch IPS practice (31). Although, we need to be careful in our conclusions based on these statistics, in which details on population characteristics including diagnosis, age and health care needs are missing, our findings are encouraging, particularly considering the higher needs of those living in supported housing (1, 4–7, 10–12).

### Interpretation of the Results

Our results suggest that IPS, an evidence-based vocational intervention (15, 17–19), is as feasible and as effective when implemented within in supported accommodation services and mental health treatment organizations in The Netherlands. This is an important finding, considering that so few clients of supported housing have competitive employment (5). Data from the current study indicates that IPS provided in supported housing can support a substantial number of clients, willing to participate in an IPS program, to find a competitive job or education.

Although IPS was originally developed for mental health treatment services (25), by analyzing the IPS fidelity assessors' reports and through staff focus groups, we were able to further understand the facilitators and barriers in implementing IPS in supported housing settings. It should be noted when considering the differences between mental health care and supported housing services on the fidelity items that the baseline fidelity measures we compared were conducted at different time over a 4 year period (from 2015 to 2019) and over this timeframe both IPS and the services within which it was implemented have developed in terms of organizational structures, quality of IPS training and quality of fidelity assessments and this may have influenced our findings. Participants of the focus group reported that the smaller IPS caseload size in supported hosing services was an important facilitator of successful job searching as their clients needed more intensive support with this.

An important difference between the sectors was the integration of the IPS workers within the service, which was lower in supported housing services. This was due to the fact that these workers were often working across multiple supported hosing sites whereas IPS workers in mental health treatment organizations were usually embedded within a single team. However, the overall fidelity rating and employment outcomes were similar for both sectors suggesting that it can be successfully implemented despite differences in specific aspects of fidelity.

Despite the positive results, the data suggest that both mental health treatment and supported housing services score, on average, “fair” on the IPS fidelity scale in their first audit. This leaves room for improvement in both sectors which could lead to greater success in employment outcomes (27). It is known from the national data set that fidelity increases in time and currently fidelity scores in both sectors are above 100, indicating good implementation. Our results also give indications as to how further improvements could be made and suggest that exchange of experiences and expertise between supported housing and mental health treatment organizations on working with IPS is potentially helpful. The main advantage of providing IPS in both sectors is the expansion of the model to a greater number of people with severe mental illness with the associated benefits of facilitating people's access to competitive employment which in turn, improves societal integration and many aspects of well-being (8, 9).

Further research is needed to understand how best to integrate IPS workers in non-clinical settings and how to ensure good liaison between sectors. This is relevant to ensure that if the IPS model expands to other sectors, the critical features are preserved and assessed. There is also more to learn from supported housing services about how they succeed in rapid job searching and engaging a diverse range of employers in the program.

Finally, the results of the focus group indicate that ongoing funding for IPS is needed. Although, in the Netherlands, currently a subsidy scheme is available for IPS, some IPS coordinators participating in the focus group experienced difficulties in accessing funding for all supported housing clients who wished to engage with IPS.

### Limitations and Strengths

In interpreting the results, it must kept in mind that the analyses were performed on a small number of IPS programs. IPS in supported housing services is a new development and the results may therefore not be generalizable. Another important limitation is the fact that we were not able to distinguish differences in features of the sample of clients that received IPS between both sectors; as our data were collated at the program level, individual client characteristics were not available. Differences in features of the sample might have been an additional indicator in the description of differences between mental health and supported housing, although in both sectors the population concerns persons with serious mental health problems and research indicates a large overlap as 80% of the supported housing clients receive mental health treatment. The main difference is that supported housing clients have higher care needs on housing and daily living, and a smaller proportion has paid work (22), which suggests that realizing paid employment might even be more difficult in this group In that light, our findings are more positive than would have been expected. An obvious strength is that this is internationally the first study providing indications on the feasibility, experiences, and effects of IPS provided in supported housing services.

## Data Availability Statement

The raw data supporting the conclusions of this article will be made available by the authors, without undue reservation.

## Ethics Statement

Ethical review and approval was not required for the study on human participants in accordance with the local legislation and institutional requirements. The patients/participants provided their written informed consent to participate in this study.

## Author Contributions

DR wrote the manuscript, performed the focus group, the qualitative analysis, and co authored the quantitative analyses. LW, CB, CC, PM, HK, and JW were involved in the outline and reasoning of the manuscript and critically reviewed the manuscript. JW is also the director of Phrenos, coordinating the IPS audits, and quantitative data collection. LW performed the quantitative analyses and the qualitative analyses. All authors contributed to the article and approved the submitted version.

## Conflict of Interest

The authors declare that the research was conducted in the absence of any commercial or financial relationships that could be construed as a potential conflict of interest.
